# Lion Brewery

**Published:** 1889-07

**Authors:** 


We invite special attention to the advertisement headed *Lion Brewery*, addressed to the medical profession, and referring to a beer adapted to *Invalids*.

